# Putting Your Money Where Your Mouth is: Examining Metacognition in ASD Using Post-decision Wagering

**DOI:** 10.1007/s10803-019-04118-6

**Published:** 2019-07-10

**Authors:** Katie L. Carpenter, David M. Williams, Toby Nicholson

**Affiliations:** School of Psychology, Keynes College, University of Kent, Canterbury, Kent CT2 7NP UK

**Keywords:** Autism, Metacognition, Mindreading, Post-decision wagering

## Abstract

It has been argued that metacognition and mindreading rely on the same cognitive processes (Carruthers in The opacity of mind: an integrative theory of self-knowledge, Oxford University Press, Oxford, 2011). It is widely accepted that mindreading is diminished among individuals diagnosed with autism (Brunsdon and Happé in Autism 18(1):17–30, 2014), however, little is known about metacognition. This study examined metacognition in relation to mindreading and autism using post-decision wagering. Results from a student sample showed negative associations between autism traits and metacognitive accuracy, and metacognitive reaction times and mindreading. These findings were replicated in a general population sample, providing evidence of a reliable association between metacognition, mindreading and autism traits. However, adults diagnosed with autism showed equivalent levels of metacognitive accuracy to age- and IQ-matched comparison participants, albeit only with an overall increase in meta-level processing time.

Autism spectrum disorder (ASD) is a developmental condition characterised by the early onset of behavioural difficulties in social communication, and restricted/repetitive behaviour and interests (American Psychiatric Association 2013). It is widely reported that, at the cognitive level, mindreading (the ability to attribute mental states to others; also known as theory of mind or mentalising) is diminished among individuals diagnosed with ASD (Brunsdon and Happé 2014). However, there is relatively little research focusing on metacognition (the ability to attribute mental states to one’s self) among individuals with autism (Carruthers 2009).

This relative lack of research into metacognition in ASD is surprising for several reasons. First, from a practical/clinical perspective, metacognition plays a vital role in everyday self-regulation (especially in education/work settings; Hacker et al. 2008; Nelson and Narens 1990; Schunk 2008), allowing one to control their thoughts and behaviour efficiently. For example, knowing that we do not know something should lead us to communicate our uncertainty (Bahrami et al. 2010), or seek out new information (Metcalfe and Finn 2008; Metcalfe 2009). These skills are important when it comes to real world situations, such as those faced in education or work. In these situations, uncertainty may lead one to revise more for an exam or to ask their supervisor for more guidance so that they can perform their job successfully. This is particularly relevant for understanding ASD, given that people with this disorder tend to have difficulties with self-regulation (Gomez and Baird 2005; Jahromi et al. 2013), under-achieve in education relative to what would be predicted based on general intelligence (Ohtani and Hisasaka 2018), and struggle to maintain long-term employment (Hendricks 2010; Shattuck et al. 2007).

Second, from a theoretical perspective, there remains a debate concerning the underlying cognitive processes involved in mindreading and metacognition. On the one hand, it has been proposed that mindreading and metacognition rely on the same neurocognitive mechanism, and therefore metacognition (as well as mindreading) should be impaired in individuals with autism (Carruthers 2011). However, others have argued that mindreading and metacognition rely on distinct processes (Nichols and Stich 2003). Given that mindreading is known to be diminished among individuals with autism,^1^ the study of metacognition in ASD has the potential to contribute to theory-building in this area. For example, if mindreading and metacognition rely on the same neurocognitive mechanism it would be predicted that there would be a significant relation between individuals’ performance on mindreading and metacognition tasks. The argument of shared mechanisms would further be supported by evidence of diminished metacognitive ability among individuals with autism. If, however, a dissociation is found, this would oppose the argument that mindreading and metacognition rely on the same processes and support the theories that suggest distinct or additional processes are at work.

Metacognition is assessed traditionally by asking individuals to make some form of judgement about their ability/performance. The closer the correspondence between a person’s judgement of their ability and their actual ability, the better a person’s metacognitive monitoring ability. Probably the most frequently used task is the classic Judgement of Confidence (JoC) task. In this task, participants make a cognitive-level (or “object-level”) judgement and then rate how confident they are that they have performed accurately. The extent to which participants’ confidence in the accuracy of their response corresponds to the actual accuracy of their response indicates their metacognitive accuracy.

To date, five studies have examined JoC among children/adolescents with autism, four of which reported diminished metacognitive accuracy (Wilkinson et al. 2010; McMahon et al. 2016; Williams et al. 2018; Grainger et al. 2016), and one of which reported no significant between-group differences (Wojcik et al. 2011). A further four studies have explored metacognition among adults with ASD, three finding metacognition to be undiminished (Wilkinson et al. 2010; Sawyer et al. 2014), one producing mixed results (Cooper et al. 2016), and one reporting a significant diminution of JoC accuracy among participants with ASD (Nicholson et al. 2019). From this limited number of studies, it is difficult to draw any firm conclusions relating to metacognition as measured by JoC in adults with autism. One possibility is that metacognitive deficits in childhood are resolved by adulthood. Another possibility is that methodological (or other) issues mask underlying deficits among adults with autism. Sawyer et al. for example, did not match groups for age or IQ. Furthermore, Cooper et al. suggest that their mixed results may have been due to the differences in object-level tasks rather than true metacognitive differences. Given these mixed results, further research is required to rectify the methodological issues and examine metacognition using object-level tasks where individuals with autism do not have specific deficits.

A more general issue to consider when interpreting results from studies of JoC accuracy in ASD is that such tasks rely on verbal reports of confidence. One potential difficulty with such verbal measures is that they rely on a subjective interpretation of “confidence”, which may vary across individuals in a way that is not measured in traditional JoC tasks (Sandberg et al. 2010). Although there are good reasons to employ verbal tasks as measures of metacognitive ability (see Nicholson et al. 2019), it would beneficial to explore other types of tasks to avoid over-reliance on a single measure. An alternative measure of metacognition that has never been employed among individuals with ASD to our knowledge, is post-decision wagering (PDW). PDW is a tangible measure and has been used as an alternative to making verbal judgements of confidence in studies involving adults and children (Ruffman et al. 2001; Persaud et al. 2007). PDW is similar to JoC in that participants are required to make a cognitive-/object-level judgement, but instead of rating their confidence they place a bet on the accuracy of their judgement. The extent to which participants make higher wagers for correct responses and lower wagers for incorrect responses is taken to indicate their metacognitive ability. Research has also shown PDW to be as effective at measuring metacognition, providing that the impact of risk aversion is controlled for (Dienes and Seth 2010). Risk aversion has been linked to the way that individuals wager regardless of their level of confidence. For example, individuals may wager low to avoid large losses regardless of their level of confidence. To address this, we included a standard measure of risk aversion in the current study.

To date there are no published studies using PDW to examine metacognition in relation to ASD. Given the potential benefits of PDW, the current study used a classic PDW task to investigate metacognition, and its relation to ASD and mindreading, in adult populations. In Experiment 1, we adopted an individual differences approach and explored the relations among metacognition, mindreading, and ASD traits in the general population. In Experiment 2, we employed a case–control design, to investigate metacognition and mindreading among adults with autism, as well as typically developing (TD) adults matched for age, IQ, and risk aversion. We used both metacognitive accuracy and metacognitive reaction times as measures of metacognition. Using metacognitive reaction times alongside metacognitive accuracy allows us to examine if individuals with autism/more autism traits take longer to make their metacognitive decisions. It is important to use both measures because, whilst adults with ASD may be equally as accurate, it is possible they are slower at making meta-level decisions. If there is a difference in metacognitive reaction times (independent of “object-level” reaction times) then it is possible that individuals with autism are using more effort and/or using a different process to reach levels of accuracy equal to TD individuals (Williams et al. 2009; Frith 2013; Bowler 1992). Based on previous research and in line with the one mechanism account, we predicted that metacognitive accuracy and metacognitive reaction times (i.e., average time taken to make a PDW, as an indicator of uncertainty) would be associated significantly with both number of ASD traits (higher ASD traits = lower accuracy and slower reaction times) and mindreading ability (higher mindreading = higher accuracy and faster reaction times), independent of perceptual/object-level accuracy and reaction times.

## Experiment 1: Method

### Participants

Thirty-nine students (30 females) from the University of Kent took part in the experiment. Participants had a mean age of 19.10 years (*SD* 0.85; range = 18–21). Participants received course credits in partial fulfilment of their degree. Both experiments in the current article were ethically approved by the University of Kent’s Psychology Research Ethics Committee (201715120681034775) and informed consent was obtained prior to commencing the tasks. All participants were debriefed following each session.

### Materials, Procedure and Scoring

#### Wagering Task

**Fig. 1:**
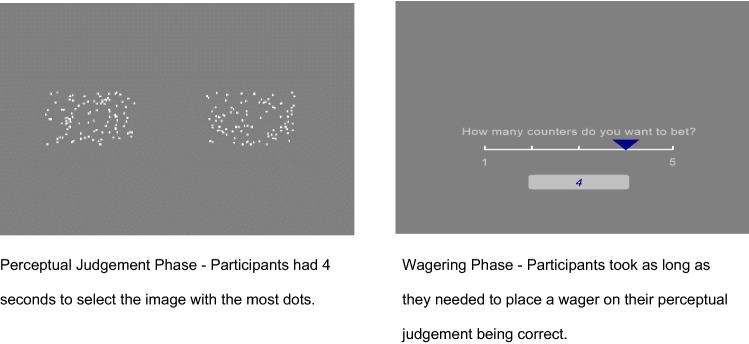
Example trial in the wagering task

This task was implemented using PsychoPy (Peirce 2007). There were two phases to the task, the *Perceptual Judgement Phase and* the *Wagering Phase* (see Fig. 1).

#### Perceptual Judgement (Object-Level) Phase

During this phase, participants were shown a series of images made up of dots (50 trials) on a computer screen. Participants were presented with two images on each trial and asked to identify which image had the most dots by clicking on the image using the mouse. The difficulty of the perceptual discrimination varied randomly across trials. Trial difficulty was operationalised in terms of the relative difference in the number of dots present in each of the two images. For example, a trial on which image A had 95 dots and image B had 125 dots (a proportional difference of .24) would be easier than a trial on which image A had 114 dots and image B had 120 dots (a proportional difference of .05). On each trial, participants had four seconds to make their judgement. If they had not made their judgement after four seconds, the programme moved on to the next trial and the trial was counted as a “miss”. The proportion of trials on which a correct perceptual discrimination was made was used as one dependent variable. The average time it took participants to make their judgment was used as the second dependent variable. The quicker the discrimination response, the easier participants found it to make their judgement.

#### Wagering Phase

On each trial, after making their perceptual judgement, participants were asked to place a wager on their answer. Participants indicated how many counters they wished to bet by using a five-point scale. Participants were informed at the beginning of the task that if they correctly identified the image with the most dots then (a) they won back the counters they wagered plus one for every counter they wagered and (b) got to keep the counters that they did not bet. So, for example, if a participant bet three counters and their answer was correct they received the three counters they bet, plus three more and got to keep the two they had left over (thus, eight counters in total). If the participant bet three counters and their answer was incorrect, they lost the counters they wagered but got to keep the counters they had left over (i.e., if they bet three of the five counters they received two counters in total). Participants were not told how much they had won until all trials were complete. Participants could win up to 500 counters. Participants were informed that the top three people with the most counters at the end of the study would receive a prize (1st = prize worth £20, 2nd = prize worth £10 and 3rd = prize worth £5). Prior to commencing the trials participants completed 10 practice trials.

“Meta-level” performance was indexed in each participant by calculating a gamma correlation (Kruskal and Goodman 1954) between perceptual discrimination accuracy and number of counters wagered, providing a measure of metacognitive accuracy. This measure has been recommended by Nelson (1984), and Nelson et al. (2004) and has been extensively used in research on metacognitive monitoring processes (e.g. Grainger et al. 2016; Sawyer et al. 2014; Williams et al. 2018). Use of gamma in the current study also serves to facilitate comparisons with other studies of metacognition in ASD, which have almost exclusively employed gamma as the main dependent variable. Metacognitive accuracy ranges from − 1 to + 1 with scores of 0 indicating chance level accuracy, and large positive scores indicating good metacognitive accuracy. “Meta-level” performance was also indexed by the average time it took for participants to place their bet across trials. The quicker the wagering response, the easier participants found it to make their judgement. One participant was excluded from all analysis because there was no variation in their wagers across trials and so a gamma score could not be calculated (leaving n = 39).

### Background Measures

Autism-spectrum Quotient (AQ; Baron-Cohen et al. 2001a). The AQ is a widely-used and well-validated self-report measure of ASD traits. It is considered to be a reliable measure of ASD traits in both clinical and subclinical populations. The AQ presents participants with 50 individual statements (e.g., “I find social situations easy”) and participants were asked to decide the extent to which they agreed with each statement by responding on a 4-point Likert scale, ranging from “definitely agree” to “definitely disagree”. Higher scores indicate more ASD traits, with a maximum possible score of 50.

Reading the Mind in the Eyes Task (RMIE; Baron-Cohen et al. 2001b). The RMIE task is a widely used measure of mindreading among intellectually able adults, including those with ASD. The task involves looking at photographs of eyes and deciding what the person in the picture is feeling. Participants were presented with 36 eye stimuli and were required to select an emotion that best described what the person in the picture may be feeling out of four possible emotions. Scores ranged from 0 to 36 with higher scores indicating better mindreading abilities.

It should be noted that the RMIE has been characterized reasonably as a kind of empathy/emotion recognition task, rather than a mindreading task specifically (see Oakley et al. 2016; but also see Nicholson et al. 2018). Yet, the task requires participants to select the most appropriate mental-state descriptor to explain the expression of a target agent, which appears to be a prima facie example of mindreading. The task has been employed in over 250 studies, and shows good test–retest reliability (e.g., Fernández-Abascal et al. 2013), clearly distinguishes groups of participants with and without ASD (e.g., Wilson et al. 2014), is associated with the number of ASD traits shown by individuals in large population studies (e.g., Baron-Cohen et al. 2001b), and is correlated with other measures of mindreading even after the influence of IQ is controlled statistically (e.g., Jones et al. 2018). Nonetheless, we appreciate the alternative views of the basis of the RMIE task and also that mindreading is a multi-faceted ability that may not be tapped by any single task. Therefore, we included additional measures of mindreading in both experiments 1 and 2.

Animations task (Abell et al. 2000). The Animations task has been widely used to assess mindreading abilities in both the general population and those diagnosed with ASD. During this task, participants were required to watch four short video clips of two triangles moving around. The clips were presented on a computer screen and, after watching each clip, participants were asked to describe what they thought was happening in the video. Participants were allowed to watch each clip twice and responses were recorded using an audio recorder and later transcribed. Accurate responses required participants to attribute mental states, such as desire and intention, to the two triangles. Scores ranged from 0 to 2 for each clip, with higher scores indicating better mindreading abilities. Participants completed one practice trial prior to commencing the test trials. Inter-rater reliability across all clips was excellent according to Cicchetti’s (1994) criteria (intra-class correlation = .89).

### Risk Aversion tasks

Lottery questions (Dienes and Seth 2010). Participants were asked two lottery questions that were as follows:

- If there was a lottery for a £10 prize, which will be given to one of the 10 ticket holders, how much would you pay for a ticket?
- If the prize were £100, which will be given to one of the 10 ticket holders, how much would you pay for a ticket?

The smaller the amount an individual is willing to pay the lesser the individual’s propensity for risk, with an optimal score of 11 indicating no risk aversion. The lottery score for our sample was not significantly different from 11, indicating that our sample was not risk averse, t(37) = .04, *p *= .972.

Balloon Analogue Risk Task (BART; Lejuez et al. 2002). The BART is a computer-based task designed to measure risk propensity. In this task participants were required to inflate a computer-simulated balloon by pressing the space bar. In the current study, participants earned virtual money with each pump, which was later converted into points (£1 = 1 point) and added on to their score on the wagering task. The amount earned in each trial was displayed on the screen with the total amount earned being presented throughout. When the balloon was pumped up too much, resulting in it exploding, participants did not gain anything for that trial. Participants were able to cease pumping the balloon at any point and bank the gains earned for that trial adding it to the total earnings. There were 20 trials in total. The smaller the average score for unexploded balloons the lower the individual’s propensity for risk. Descriptive statistics for each of the background and risk measures are presented in Table 1.

**Table 1:** Means and standard deviations for background and risk measures in Experiment 1

Variable	Mean	SD	Range
Autism Quotient	16.31	6.22	2–27
Animation	6.34	1.44	2–8
Reading the Mind in the Eyes	25.62	5.11	16–34
Balloon Analogue Risk Task	21.85	10.71	5–39
Lottery	11.05	9.18	0–55

### Statistical Analysis

Reported significance values are for two-tailed tests. However, when results are predicted a priori on the basis of a solid theoretical foundation and/or previous empirical findings, it is arguably not only legitimate to use one-tailed tests, but also sensible to do so (see Cho and Abe 2013). In the current study, predictions were entirely in keeping with those made in our previous work on this topic and with published findings. Therefore, in instances where explicitly predicted results were non-significant when reported using two-tailed tests, but significant (or very close to being significant) when used one-tailed tests, we report the results from both. Where t-tests were used, we report Cohen’s *d* values as measures of effect size (≥ .0.20 = small effect, ≥ 0.50 = moderate effect; ≥ 0.80 = large effect; Cohen 1969). Where ANOVAs were used, we report partial eta squared (ƞ_*p*_^2^) values as measures of effect size (≥ .01 = small effect, ≥ .06 = moderate effect, ≥ .14 = large effect; Cohen 1969).

## Experiment 1: Results

Descriptive statistics for the wagering task are presented in Table 2. The gamma score on the wagering task was significantly different from zero, *t*(38) = 5.50, *p *< .001, indicating that participants were significantly above chance in their wagering accuracy, placing higher bets for correct answers than for incorrect answers. Table 3 shows non-parametric correlations among the key variables.^2^

**Table 2:** Means and standard deviations for the wagering task in Experiment 1

Variable	Mean	SD	Range
Object-level proportion correct	.66	.07	.56 to .80
Missed trials	1.10	1.47	0 to 5
Object-level reaction times (s)	1.87	0.39	1.20 to 2.64
Counters wagered	2.78	0.71	1.00 to 4.44
Wagering reaction times (s)	1.60	0.39	1.00 to 2.83
‘Meta-level’ gamma	.29	.33	− .43 to 1

**Table 3:** Correlations between key variables in Experiment 1

Variables	2	3	4	5	6	7	8	9	10
1. Object-level proportion correct	− .30	− .19	− .36*	− .01	− .29	.19	.40*	.14	− .38*
2. Counters wagered		− .04	.02	.04	.17	− .07	− .18	.15	.06
3. Object-level reaction times			.55**	.22	− .13	− .14	.02	.13	.32
4. Wagering reaction times				.11	.17	− .18	− .33*	.04	.30
5. ‘Meta-level’ gamma					− .32*	.13	− .04	− .05	.27
6. Autism Quotient						− .16	− .21	− .24	.20
7. Animation							.43**	− .21	.01
8. Reading the Mind in the Eyes								− .20	− .26
9. Balloon Analogue Risk Task									− .16
10. Lottery									

*p < .05, **p < .01

As predicted, AQ score was significantly negatively associated with gamma. Partial correlation analysis showed that this association remained significant even after controlling for proportion of correct object-level discriminations, *r*(36) = − .34, *p *= .04. In contrast to what was predicted, AQ score was not significantly related to wagering reaction times (RT; seconds). In terms of mindreading, RMIE was significantly negatively associated with wagering RT. This correlation remained significant even after controlling for object-level RT, *r*(36) = − .41, *p *= .01.

## Experiment 1: Discussion

As predicted, the results from Experiment 1 showed that there was a significant relation between metacognitive accuracy and ASD traits, indicating that the more ASD traits an individual had the less accurate they were in their metacognitive judgements. Unexpectedly, there was no significant relation between mindreading ability and metacognitive accuracy. However, as predicted, there was a significant relation between wagering RT and mindreading as measured by the RMIE task. The better the participant’s mindreading ability, the *quicker* they made their wagering judgements, independent of object-level RT. This implies that those with better mindreading skills are able to access metacognitive processing and interpret it quicker, and thus arrive at a wagering decision with relative ease. It should be noted, however, that wagering RT was non-significantly associated with performance on the Animations task. This could be due to the relatively limited variance in scores on the Animations task (0–8, rather than 0–36 on the RMIE task) masking an underlying association. To address these issues in Experiment 2, we employed a measure of mindreading with a wider range of scores than is possible on the Animations task (the Movie for the Assessment of Social Cognition; Dziobek et al. 2006). From these results, it was predicted that the ASD participants would show significantly lower wagering accuracy and significantly longer wagering RT than TD participants in Experiment 2.

## Experiment 2: Method

### Participants

Twenty-one adults with a diagnosis of ASD (13 males) and 20 TD (14 males) adults took part in the current study. All of the participants in the ASD group had received a formal diagnosis of ASD in accordance with established criteria (American Psychiatric Association 2013; World Health Organization 1993).

Details of participant characteristics can be seen in Table 4. Full Scale (FSIQ), Verbal (VIQ) and Performance (PIQ) IQ were assessed using the Wechsler Abbreviated Scale for Intelligence-II (Wechsler 1999). All participants also completed the AQ as a measure of ASD traits and the BART as a measure of risk aversion. Thirty-nine participants also completed the Lottery questions; the remaining two (1 ASD, 1 TD) did not due to administration error. Participants in the ASD group also completed the Autism Diagnostic Observation Schedule, a semi-structured observational measure of ASD features (Lord et al. 2000). Finally, all participants completed two measures of mindreading ability, the RMIE task and the Movie for the Assessment of Social Cognition (MASC; Dziobek et al. 2006), which is described in detail below. There were no significant differences between the ASD and TD group in terms of age, propensity for risk, FSIQ, VIQ, or PIQ. There were, however, expected between-group differences in number of ASD traits (in line with their diagnostic status) and mindreading ability. Informed consent was obtained prior to commencing the tasks. All participants received payment of £7.50 per hour for their time and travel expenses, and all participants were debriefed following each session.

**Table 4:** Experiment 2 participant characteristics: means, standard deviations (in brackets), and inferential statistics

	Group		*t*	*p*	Cohen’s *d*
	ASD (n = 21)	TD (n = 20)			
Age	36.86 (12.22)	41.95 (13.94)	− 1.25	.22	0.39
Full-scale IQ	105.62 (13.18)	105.65 (12.99)	− 0.01	.99	< 0.01
Range	73–122	83–132			
Performance IQ	106.14 (16.87)	105.60 (15.18)	− 0.09	.93	0.04
Range	65–132	76–141			
Verbal IQ	105.38 (11.45)	104.05 (11.22)	0.38	.71	0.12
Range	86–128	81–129			
Autism Quotient	33.00 (8.20)	14.25 (4.56)	8.99	< .001	2.82
Reading the Mind in the Eyes	24.95 (5.35)	27.80 (3.86)	− 1.95	.06	0.61
Movie for the Assessment of Social Cognition—Total	28.10 (6.58)	33.75 (5.21)	− 3.04	< .001	0.95
Movie for the Assessment of Social Cognition—Control	3.43 (1.29)	4.40 (1.06)	− 2.60	.01	0.81
BART	20.17 (9.24)	25.46 (12.36)	− 1.56	.13	0.48
Lottery	11.89 (24.14)	5.97 (4.30)	1.01	.29	0.36

### Materials, Procedure and Scoring

Participants completed the AQ, RMIE, BART, lottery and wagering task all of which are described above. The procedures for AQ, RMIE and lottery were the same as in Experiment 1, although the BART involved earning money instead of points in Experiment 2. Participants also completed the MASC where they watched a short film of a group of people interacting. The film was stopped at regular intervals and the participant was asked a question about what the person in the film was thinking or feeling at the moment the film was stopped. Each question was multiple choice and participants were presented with four answers to choose from. The higher the score on the MASC the better the individual’s mindreading abilities. The MASC also includes six control questions that require mental flexibility and abstract reasoning without any demand on social-cognitive competencies.

The wagering task had a similar procedure and scoring method as that used in Experiment 1, with only slight differences in each phase. In the *Judgement Phase,* approximately half of the participants in each group completed the same perceptual discrimination task (the dots task) as participants completed in Experiment 1. However, the other half of participants in each group completed an analogous task that involved choosing on each trial which of two lines was longest (rather than which of two boxes had the most dots in). The reason for this is that some participants had already completed the dots task as part of another study in our lab. To ensure there were no systematic differences between tasks across groups, an initial two-way ANOVA was conducted. Main effects showed that there was a significant main effect of task version, *F*(1,37) = 5.22, *p *= .03, ƞ^2^ = .12, but not group, *F*(1,37) = .38, *p *= .54, ƞ^2^ = .01. The task main effect indicates that participants who took part in the lines version correctly discriminated a higher proportion (.71) in comparison to the dots task (.65). Crucially, the analysis confirmed that there was no significant Group × Task version interaction on the proportion of stimuli correctly discriminated, *F*(1,37) = 0.11, *p *= .72, ƞ^2^ = .003.

In the *Wagering Phase*, the only difference in procedure in Experiment 2 from that in Experiment 1 was that money was offered instead of prizes. Hence, in Experiment 2, the number of counters participants bet was equal to the number of pennies they wish to bet, 1 counter = 1p, 2 counters = 2p and so on. One participant (with ASD) was excluded from all analysis because there was no variation in the amount they wagered across trials and so a gamma score could not be calculated. This resulted in a final ASD sample of n = 21.

## Experiment 2: Results

With regard to object-level performance, there were no significant differences between participants with ASD (M = .67, SD = .08) and comparison participants (M = .68, SD = .09) in the proportion of trials on which stimuli were correctly discriminated, *t*(39) = − 0.44, *p *= .66, *d *= 0.12. Moreover, there was no significant difference between the ASD group (M = 2.09, SD = .44) and TD group (M = 2.02, SD = .42) in the average number of seconds to make their perceptual judgement during the object-level phase, *t*(39) = 0.48, *p *= .64, *d *= 0.16. Thus, the two groups were very similar with respect to cognitive-/object-level ability (accuracy *and* speed).

In the wagering phase, there was no significant difference between the ASD group (M = 3.03, SD = .96) and TD group (M = 3.31, SD = 1.08) in number of counters wagered, *t*(39) = − .91, *p *= .37, *d *= 0.27. This confirms the findings from the BART and lottery tasks (see Table 2) that participants with ASD were not inherently more risk averse than comparison participants. Unexpectedly, the mean gamma score among participants with ASD (M = .37, SD = .26) was non-significantly smaller than the gamma score among TD participants (M = .44, SD = .29), *t*(39) = − .76, *p *= .45, *d *= 0.25. However, as expected, the mean wagering RT was significantly longer among participants with ASD (M = 2.09, SD = .43) than among TD participants (M = 1.83, SD = .36), *t*(39) = 2.08, *p *= .04, *d *= 0.66. This remained significant (and increased somewhat in magnitude) after controlling for object-level RT, *F*(1,38) = 6.70, *p *= .01, ƞ_*p*_^2^ = .15.

### Correlations

To examine the relationship between ASD traits (AQ), metacognition and mindreading (RMIE and MASC) a series of correlational analyses were conducted among each group. In the ASD group, there were no significant correlations between wagering RT or gamma, and mindreading or ASD traits, all rs < .28, all ps > .22. However, in the TD group, results replicated closely those observed in Experiment 1.

There was a negative correlation between AQ score and gamma among TD participants, *rs*(19) = − .42. This correlation was close to statistical significance when using a two-tailed test, *p *= .07 and statistically significant when using a one-tailed test, *p* < .04 (which is arguably legitimate, given that it was a predicted effect). In this context, it is important to note that this correlation is actually slightly stronger than the AQ score × gamma correlation observed among TD participants in Experiment 1 (*r *= − .32 in Exp. 1 vs *r* = − .42 in Exp. 2), albeit non-significantly so according to Fisher’s Z test, *z *=0.39, *p *= .35. This suggests that the failure to reach conventional levels of statistical significance (when using a two-tailed test) was the result of the lower statistical power in Experiment 2 than in Experiment 1. Likewise, after controlling for object-level performance (proportion correct), the AQ score × gamma correlation in Experiment 2 was non-significant when using a two-tailed test, *rs*(17) = − .37, *p *= .12, but marginally significant when using a one-tailed test, p = .06. Again, the partial AQ score × gamma correlation in Experiment 2 was slightly stronger than the equivalent partial correlation in Experiment 1 (*r *= − .34 in Exp. 1 vs *r* = − .37 in Exp. 2). All other analyses examining the relationships between gamma scores and mindreading for the TD group were non-significant, all *rs *< .17, all *ps *> .24.

In terms of wagering RT, among TD participants, there was a significant negative correlation between wagering RT and performance on the MASC, *rs*(18) = − .73, *p *< .001. This remained significant when controlling for object-level RT, *rs*(17) = − .73, *p *< .001 and proportion correctly discriminated, *rs*(17) = − .69, *p *< .001. All other analyses examining the relationships between wagering RT, and mindreading (RMIE) and ASD traits were non-significant, all *rs *< − .14, all *ps *> .55.

Due to the relatively small sample sizes across the two experiments we combined the student sample from Experiment 1 and the TD sample from Experiment 2 (*n *= 59) to increase statistical power. Post hoc analysis using G*Power 3.1 (Faul et al. 2007) revealed the statistical power for detecting a medium effect size (.3) for the combined samples was .77. The combined sample analysis revealed that the significant negative correlation between AQ score and gamma (*rs*(59) = − .32, *p *= .01) remained significant when controlling for object-level performance (proportion correct), *rs*(56) = − .31, *p *= .01. There remained no significant correlation between gamma and RMIE, *rs*(59) = .05, *p *= .36. Combining the data also showed that there was a marginally significant negative relationship between wagering RT and RMIE, *rs*(59) = − .21, *p *= .06 (which was significant of reported using a one-tailed test, *p* = .03), but the relationship between wagering RT and AQ score for the combined samples remained non-significant, *rs*(59) = .10, *p *= .22.

## Experiment 2: Discussion

The results from Experiment 2 revealed that there was no significant difference in metacognitive accuracy between the ASD group and the TD group, in contrast to what was predicted. There was, however, a significant between-group difference in meta-level reaction time. This suggests that the ASD group may be using a different process, which requires additional processing time, to reach the same level of metacognitive accuracy as the TD group. The significant association between metacognitive accuracy and autism traits found in Experiment 1 was replicated among the TD group in Experiment 2. Furthermore, the relationship between mindreading and meta-level reaction times found in Experiment 1 was replicated in the TD group (as measured by the MASC). This suggests that individuals with poorer mindreading abilities took longer to make a metacognitive decision.

## General Discussion

To our knowledge, this is the first study to investigate metacognition in relation to ASD and mindreading using PDW. The key results were that ASD traits were significantly related to metacognitive accuracy (more ASD traits = lower accuracy) and mindreading ability was associated significantly with metacognitive RT (better mindreading = faster RTs). These results, which we interpret below, should lead to the prediction that adults with a full diagnosis of ASD would show impairments in *both* measures of metacognitive performance (accuracy and RTs). In keeping with this prediction, wagering RTs were significantly longer among ASD participants than among TD participants in Experiment 2. In both experiments, these significant associations with meta-level performance (RTs and accuracy), were independent of the influence of object-level performance, showing the associations are specific to *meta*cognitive, rather than cognitive, processes. In other words, it was not the case that decision-making, motor co-ordination, or general speed of processing were relatively slow among ASD participants, rather that *meta*cognitive decision-making specifically was diminished in this sample. Perhaps most important, this pattern of associations was found in independent samples of TD adults across two experiments, which provides reassurance about the reliability of results.

Contrary to our prediction, however, there was no evidence of an ASD-specific impairment in metacognitive accuracy in Experiment 2. The between-group difference in wagering accuracy was non-significant and associated with only a small effect size (*d *= 0.24). This is puzzling, given the reliable association between the number of ASD traits and metacognitive accuracy in the general population. Logically, if we find a relation between variables A and B in a sample of individuals with high/clinically-significant ASD traits, then this might not necessarily hold among people with lower ASD traits, or for the general population in which high AQ scores are apparent in a small proportion of individuals. However, if the A–B correlation is reliable in the general population (which it is in our study), then it should hold in diagnosed individuals who have high ASD traits by definition. There are two possible explanations for this pattern of results, as far as we can deduce.

First, it could be that Experiment 2 was underpowered and that a larger sample of participants would have yielded a significant between-group difference in metacognitive accuracy. This is possible, of course. The sample of ASD participants was not large (which is true of many studies in the field) and so the study was not sufficiently powered to detect small/modest between-group differences. Clearly, however, the sample *was* sufficiently powered to detect significant between-group differences in metacognitive RTs (which were moderate in size; *d *= 0.65) as well as a significant association between the number of ASD traits and metacognitive accuracy among TD participants. Thus, while it may be that a larger sample would have revealed a deficit in metacognitive accuracy among individuals with autism, such a deficit would not likely be as marked as the observed deficit in metacognitive RTs and, thus, not of clinical significance, potentially.

A second explanation for the current findings is that wagering accuracy is undiminished in ASD, but underpinned by slower processing efficiency in this domain which increases the amount of time people with this disorder need to make accurate metacognitive judgements. While this is a possible explanation for some of the findings, it does not appear to explain the results from the correlation analyses in Experiment 2. If performance on the wagering task was underpinned by the same underlying metarepresentational/metacognitive resources in each diagnostic group, but just resources that are accessed less quickly/efficiently among ASD than comparison participants, then associations among measures should be of a similar magnitude in each group. Yet, this was not the case. Among TD participants, wagering accuracy was associated significantly negatively with number of autism traits (r = − .42), but this did not hold up among participants with ASD (r = .07). Likewise, wagering reaction time was associated significantly with performance on the MASC measure of mindreading among TD participants (r = − .73), but not among participants with autism (r = − .19). The different patterns of association among measures in each diagnostic group suggests that the underlying processing resources used to arrive at accurate wagering decisions was different in each group. Therefore, we believe that a third explanation is more plausible, namely that participants with autism were using alternative, possibly compensatory, strategies to perform well in terms of metacognitive accuracy despite limited underlying metarepresentational competence (see Livingston and Happé 2017).

In other domains, it has been shown that individuals with autism use alternative strategies to perform well on tasks despite possessing atypical underlying conceptual competence (Bowler 1992; Hermelin and O’Connor 1985). This explanation fits well with evidence that adults with autism tend to rely on deliberative reasoning strategies to solve cognitive tasks, rather than relying on intuitive processes employed by TD adults (Brosnan et al. 2016). According to Dual-Process theory (Evans and Frankish 2009), human decision-making is underpinned by two forms of reasoning. Reasoning based on heuristics (non-analytic) tend to be fast, easy and intuitive (Type 1) and reasoning based on analytic processes tend to be slower, more effortful, and deliberative (Type 2). This notion fits well with the current findings and may also explain previous findings in the literature. Whereas TD adults from the general population tend to employ type 1 reasoning when completing metacognitive monitoring tasks, adults with autism tend to employ type 2 reasoning, which results in similar levels of accuracy but after a longer period of processing. This explains how the reliable association between ASD traits and metacognitive accuracy in the general population did not hold in the ASD sample in Experiment 2; the association we observed was between number of ASD traits and type 1 reasoning about one’s confidence. ASD participants in Experiment 2 were using type 2 processing and this afforded them the opportunity to make accurate judgements despite their ASD. The previous mixed findings regarding accuracy of verbal judgements of confidence among adults with autism could also be explained in this way. Under some circumstances, it may be that deliberative reasoning about one’s mental states yields inaccurate judgements/behaviour (and thus between-group differences in studies of monitoring accuracy). However, mostly such reasoning will yield accurate judgements (albeit after longer processing) and so between-group differences will not be observed. One potentially important issue to consider is whether there is a developmental process at work also. Intellectually-able adults with ASD have already been through an education system that encourages the development of metacognitive skills, so arguably type 2 reasoning about mental states becomes ingrained as a response to training and difficulties with intuitive monitoring earlier in life. In the context of the current study, this hypothesis would lead to the prediction that children with ASD would show significantly diminished metacognitive accuracy on the wagering task. Understanding developmental processes and not just behavioural outcomes is crucial to expanding our understanding of cognitive functioning as a whole in ASD. From a theoretical perspective, the current results are partly in keeping with the idea that metacognition and mindreading share metarepresentational processing resources. Specifically, the speed with which one can metarepresent self (wagering reaction time) was associated specifically with the ability to metarepresent others (on the MASC and RMIE). Equally, participants with ASD showed impairments in both mindreading and metacognitive processing speed, independent of general (object-level) processing speed. These findings are consistent with the ideas that mindreading and metacognition depend on the same underlying metarepresentational resources, and that these resources are diminished in ASD causing impairments in both domains (e.g., Carruthers 2011; Williams 2010). Contrary to expectations, however, (a) wagering accuracy was non-significantly associated with mindreading ability, and (b) participants with ASD did not show diminished wagering accuracy. We discussed possible reasons for finding (b) above. Finding (a) was surprising, because two previous studies have reported a significant association between verbal judgement of confidence accuracy and mindreading abilities (Nicholson et al. 2018; Williams et al. 2018), and most assume that wagering requires the same underlying conceptual resources as judgement of confidence tasks, but just a different response mode. Of course, one possibility is that wagering accuracy relies on different underlying conceptual resources to judgement of confidence accuracy, and that only the latter requires metarepresentation (hence, only a correlation between judgement of confidence accuracy and mindreading task performance, but not between wagering accuracy and mindreading task performance). While this is possible, it does not explain why wagering reaction times were associated with mindreading ability. The wagering task must have tapped metarepresentational processing in some way, so it does not appear to be the case that it is not metarepresentational at all. Another possibility, therefore, is that a true underlying association in the current study between wagering accuracy and mindreading was masked by the different, non-metarepresentational demands of the tasks. The fact that the mindreading tasks employed in the current study had a verbal response mode, whereas the wagering task required only behavioural responses, may have influenced results. This idea could be tested in future studies by employing verbal and non-verbal measures of mindreading and metacognition to investigate whether specific associations exist between measures that have equivalent response modes.

Overall, the current research provides evidence that adults with ASD are just as accurate as TD adults at wagering on their perceptual judgements (implying undiminished metacognitive monitoring accuracy), albeit only with an overall increase in processing time. This is important given that metacognitive accuracy can have an impact on an individual’s daily functioning (Hacker et al. 2008; Nelson and Narens 1990), from basic tasks such as crossing a road to more complex tasks within the work place, or even the extent to which a jury will believe a witness statement (Cutler et al. 1988).

## Footnotes

1 The authors acknowledge that there is an ongoing debate among individuals who have been diagnosed with ASD concerning the terminology used when referring to individuals with such a diagnosis. This article uses ‘individuals with autism’, however the authors acknowledge that some individuals diagnosed with ASD prefer to be referred to as ‘autistic’. Due to the ongoing debate, the authors have chosen to use ‘individuals with autism’ in line with the majority of research literature, however it is acknowledged that ‘autistic’ may be preferable to some people and therefore the authors do not mean to cause any offence with the terminology used within this article.

2 Due to the unequal number of males and females within this sample, analysis was conducted to examine if there was any significant difference between males and females on key variables. This analysis showed that there was a significant difference for average wager (*t*(37) = 4.15, *p *< .001, *d *= 0.41), with males (M = 3.5; SD = .51) placing higher wagers than females (M = 2.6; SD = .62), and proportion correct (*t*(37) = − 2.67, *p *= .0, *d *= 0.17), with females (M = .68; SD = .06) getting a higher proportion of answers correct compared to males (M = .61; SD = .06). Analysis also showed that there was a marginally significant difference between males and females for RMIE (*t*(37) = − 1.97, *p *= .06, *d *= 0.74), with females (M = 26.47; SD = 4.89) scoring higher than males (M = 22.78; SD = 5.07). All remaining variables, including gamma, were non-significant (all *ts *< 1.05, all *ps *> .30). Analysis was also conducted to ensure that there were no significant differences in correlations between gamma scores or wagering RT and mindreading (RMIE, Animations) or Autism traits (AQ). This analysis showed that there were no significant differences between males and females for any of the correlations according to Fisher’s Z test, all zs < − 1.61, all *ps *> .11.
